# Veteran families with complex needs: a qualitative study of the veterans’ support system

**DOI:** 10.1186/s12913-021-07368-2

**Published:** 2022-01-15

**Authors:** Angela M. Maguire, Julieann Keyser, Kelly Brown, Daniel Kivlahan, Madeline Romaniuk, Ian R. Gardner, Miriam Dwyer

**Affiliations:** 1 Gallipoli Medical Research Foundation, Greenslopes Private Hospital, Brisbane, Australia; 2 Faculty of Medicine, The University of Queensland, Brisbane, Australia; 3 Department of Psychiatry and Behavioral Sciences, University of Washington, Seattle, Washington USA; 4 Faculty of Health and Behavioural Sciences, The University of Queensland, Brisbane, Australia

**Keywords:** Veterans, Family, Military, Health services accessibility, Care coordination, System integration, Trauma and stressor related disorders, Health, Social adjustment, Qualitative research

## Abstract

**Background:**

Families with complex needs face significant challenges accessing and navigating health and social services. For veteran families, these challenges are exacerbated by interactions between military and civilian systems of care, and the density of the veterans’ non-profit sector. This qualitative study was designed to gather rich, detailed information on complex needs in veteran families; and explore service providers’ and families’ experiences of accessing and navigating the veterans’ support system.

**Methods:**

The study comprised participant background questionnaires (*n* = 34), focus groups with frontline service providers (*n* = 18), and one-on-one interviews with veteran families (*n* = 16) in Australia. The semi-structured focus groups and interviews were designed to gather rich, detailed information on four study topics: (i) health and wellbeing needs in veteran families; (ii) service-access barriers and facilitators; (iii) unmet needs and gaps in service provision; and (iv) practical solutions for improving service delivery. The study recruited participants who could best address the focus on veteran families with complex needs. The questionnaire data was used to describe relevant characteristics of the participant sample. The focus groups and interviews were audio-recorded, transcribed, and a reflexive thematic analysis was conducted to identify patterns of shared meaning in the qualitative data.

**Results:**

Both service providers and families found the veterans’ support system difficult to access and navigate. System fragmentation was perceived to impede care coordination, and delay access to holistic care for veteran families with complex needs. The medico-legal aspects of compensation and rehabilitation processes were perceived to harm veteran identity, and undermine health and wellbeing outcomes. Recovery-oriented practice was viewed as a way to promote veteran independence and self-management. Participants expressed a strong preference for family-centred care that was informed by an understanding of military lifestyle and culture.

**Conclusion:**

The health and wellbeing needs of veteran families intensify during the transition from full-time military service to civilian environments, and service- or reintegration-related difficulties may emerge (or persist) for a significant period of time thereafter. Veteran families with complex needs are unduly burdened by care coordination demands. There is a pressing need for high-quality implementation studies that evaluate initiatives for integrating fragmented systems of care.

**Supplementary Information:**

The online version contains supplementary material available at 10.1186/s12913-021-07368-2.

## Background

Families with a lived experience [1] of military service (i.e., veteran families) face a variety of adjustment challenges that place them at risk for poor health and wellbeing outcomes [2–13]. These challenges are characteristic features of the military lifestyle and involve: (i) repeated disruptions to family system functioning associated with training commitments and deployment cycles; (ii) periodic displacement of employment, education, childcare, healthcare, and social networks due to posting cycles [14]; (iii) primary and secondary exposure to service-related trauma; (iv) risk of service-related injury and death; and (v) cumulative losses associated with the transition from full-time military service to civilian environments (i.e., civilian reintegration) [2–13]. There are ongoing concerns about elevated suicide risk in former-serving military personnel: in Australia, ex-serving males are 24% more likely, and ex-serving females are 102% more likely (approximately twice as likely), to die by suicide than the general population [15].

Civilian reintegration has been identified as a particularly vulnerable period for veteran families [9–13]. The veteran’s transition to civilian environments has been characterised as a dynamic process of adaptation to multiple losses including military identity, culture, employment, income, healthcare, housing, and social networks [9–13]. The losses that characterise the reintegration experience have direct and indirect effects for veterans’ family members, particularly their partners and children [11, 16]. While many contemporary veterans successfully navigate this transition; approximately 50% experience service- or reintegration-related difficulties that require low- to high-intensity intervention (i.e., self-management through to complex care coordination) [9, 17]. This paper focuses on former-serving veteran families with complex needs [18].

### Complex needs

Families with complex needs have severe, persistent, interconnected difficulties that span multiple domains of functioning [18]. Such families face significant challenges accessing and navigating the services required to address the breadth and depth of their needs [18]. In 2015, estimates of focal health and wellbeing needs in a national, stratified sample^1^ of a recently discharged cohort (2010–2015) of Australian veterans indicated: 58.7% had one or more doctor-diagnosed medical conditions; 46.4% had a formally-diagnosed mental health condition (55.5% of whom had at least one co-morbid mental health condition); 43.7% reported a period of unemployment lasting at least 3 months since discharge; 43.6% had a service-related compensation claim accepted; 31.7% were not in paid employment; 28.3% had no post-secondary education; 23.0% were not in a relationship; 20.4% had been medically discharged; 7.2% were not receiving income; 6.2% were not in stable housing (last 2 months); and 2.9% reported having been arrested, convicted (2.1%), or imprisoned (0.07%) since transition [17]. The report did not provide estimates of the percentage of the cohort with complex needs spanning the aforementioned health and socioeconomic domains of functioning.

### Systems of care

Similar to the United States, Australia’s health and social care system is relatively complex; characterised by multiple payer-provider schemes that exist across multiple levels of government and multiple sectors (e.g., the Medicare Benefits Scheme [MBS]; the Pharmaceutical Benefits Scheme [PBS]; the National Disability Insurance Scheme [NDIS]; private insurers; public compensation and rehabilitation schemes; state-funded Hospital and Health Services [HHSs]; federally-funded Primary Health Networks [PHNs]; and other public, private and non-profit agencies). For veteran families, the challenges of service navigation are compounded by the military system of care (e.g., the Department of Defence [Defence]; the Australian Defence Force [ADF]; Defence Member and Family Support [DMFS; formerly the Defence Community Organisation]; the Commonwealth Superannuation Scheme [CSC]; the Department of Veterans’ Affairs [DVA]; the Repatriation Pharmaceutical Benefits Scheme [RPBS]); and the density of the Ex-Service Organisation (ESO) non-profit sector. In 2015, more than 3000 charities and trusts were providing support to Australian veteran families; of these, over 500 entities identified veteran families as the sole beneficiaries of their services [19]. Gill et al. [20] provide a detailed summary of the interface between Australian military and civilian systems of care, and the services and supports available to veteran families across the transition pathways from military to civilian environments.

A series of recent government inquiries have identified a need to better integrate the many services and programs offered to Australian veteran families [15, 21–24]. However, very little research has examined their experiences of navigating military and civilian systems of care. One qualitative study by Muir [25], commissioned by DVA as part of a joint initiative with Defence, included a component that asked family members (i.e., partners, parents, and adult children; *n* = 25) about strategies for improving the civilian reintegration process. Family members reported a need for: (i) better discharge planning for veterans and families (predominantly more individually-tailored assistance for veterans who were medically discharging or suffering from severe/ongoing health conditions); (ii) clearer communication with families that was more targeted to their specific circumstances; and (iii) service provision that was more proactive [25].

A comprehensive understanding of the needs, values, and preferences of veteran families is required to tailor a system integration solution that can be implemented and sustained in the real-world setting [26]. This qualitative study was designed to gather rich, detailed information on complex needs in veteran families; and explore service providers’ and families’ experiences of accessing and navigating the veterans’ support system. The study is described according to the COnsolidated criteria for REporting Qualitative research (COREQ) [27].

## Method

### Design

Phenomenology was selected as the research framework for the study due to its focus on the human subjective experience: the philosophical basis of the approach emphasises participants’ lived experience of the study topic(s), views their experiences as conscious and subjective, and focuses on describing (rather than explaining) the essence of their experiences [28]. The study comprised background questionnaires, focus groups with frontline service providers, and one-on-one interviews with veteran families. The semi-structured focus groups and interviews were designed to gather rich, detailed information on four study topics: (i) health and wellbeing needs in veteran families; (ii) service-access barriers and facilitators; (iii) unmet needs and gaps in service provision; and (iv) practical solutions for improving service delivery. Veteran families were explicitly asked about the aforementioned topics with reference to the military discharge process. Service providers were explicitly asked about veteran families that they found difficult to support within the current service system. The protocols for the focus groups with frontline service providers (including the discussion guide), and interviews with veteran families, are provided in Additional files 2 and 3, respectively.

The background questionnaires were used to describe relevant characteristics of the participant sample. For service providers, the questionnaire included information on employment status, job title, current role, type(s) of service provision, professional background, experience providing veterans’ support services, and lived experience of military service. For veteran families, the questionnaire included information on demographic characteristics, family structure, brief medical and psychological history, engagement with services, and brief history of military service (self or veteran relative).

### Setting

The study targeted veterans’ support services in metropolitan areas of South East Queensland, Australia. The five study sites included the four veterans’ support organisations who participated in the study, and the Foundation commissioned to lead the research. The focus groups with service providers were conducted onsite at their place of employment during work hours. The interviews with veteran families were conducted at one of the study sites (by phone, *n* = 13, or face-to-face, *n* = 3; based on the participant’s preference) at a time that was convenient for the participant.

### Research team and reflexivity

The Foundation is an independent, non-profit charity that conducts research with veteran families and the broader Australian community. AMM is employed by the Foundation as a Clinical Psychologist and Principal Research Fellow. She has training, skills, and experience in mixed-methods research and person-centred interviewing techniques; a clinical background in complex trauma [29]; and an academic background in human learning and memory (qualifications: Research PhD in the field of Psychology; Master of Clinical Psychology; Bachelor of Arts, Honours, with a double major in Psychology). AMM did not have pre-existing relationships with the participants; nor had she previously received services from, or been employed by, the veterans’ support organisations who participated in the study.

AMM coordinated the study, screened the veteran family participants, and facilitated the focus groups and interviews. Only AMM and the participants were present during the focus groups and interviews, and no repeat groups or interviews were conducted. The groups and interviews were audio-recorded and transcribed verbatim by a professional transcription agency (encrypted upload and download to secure sites). AMM reviewed the completed transcripts while listening to the audio-recordings and correcting inaccuracies in the transcription. To ensure the rigour and reliability of the transcription, the recordings and transcripts were subsequently independently reviewed by two research assistants prior to analysis. The transcripts were not returned to participants for comment or correction.

AMM conducted the thematic analysis and interpretation. She kept a reflexive journal throughout the data collection and analysis process to identify potential sources of coding or interpretation bias. To ensure the rigour and reliability of the thematic analysis, the transcripts, codes, and themes were subsequently inductively reviewed by KB (a female psychologist employed by the Foundation), who kept a reflexive journal throughout the review process (time and resource constraints necessitated review of the coding in place of independent dual coding of the data). Discussion and re-examination of the thematic analysis were jointly conducted by AMM and KB. Some refinements were made to codes at the lower levels of the analysis (two pairs of codes were merged into single codes and renamed, and five codes were reorganised) but there were no changes to the themes at the higher levels of the analysis. The participants did not provide feedback on the findings.

The informed consent form advised participants that the research was being led by the Foundation, and that the data would be collected by a registered psychologist. Participants were provided with the name of the funding agency, and the veterans’ support organisations participating in the study. They were assured their contributions to the research would be confidential, their de-identified data would be stored on a password-protected computer in a secure area of the Foundation, and their responses would not, in any way, affect their current or future entitlements to pensions, benefits, or services.

### Participants

Purposive sampling was used to select participants who could best address the focus on complex needs in veteran families. The “gold standard” for determining sample size in qualitative studies is data saturation [30]. As data saturation can only be determined once data is collected, a sample size of five to 25 individuals is recommended for phenomenological studies [31].

Between October and December 2019, 18 frontline service providers were recruited by service managers (or equivalent) from the four veterans’ support organisations who participated in the study. Service providers were eligible to participate if they met the following criteria: (i) 18+ years of age; (ii) delivering services (paid or voluntary) to veterans and/or their family members; and (iii) a minimum of 6 months (full-time equivalent) experience working with the veteran population. The focus groups (*n* = 4) ranged in size from four to five participants.

Between October and December 2019, 21 veteran family participants were recruited by service providers at the four veterans’ support organisations who participated in the study, and the Foundation recruited five veteran family participants not engaged with these organisations. Individuals were eligible to participate if they were: (i) 18+ years of age; and (ii) a veteran or the family member of a veteran. They were excluded from participating if they met any of the following criteria at the time of screening: (i) currently serving in the military; (ii) inpatient on a psychiatric ward; (iii) recent (i.e., last 3 months) emergency department presentation or hospital admission for the treatment of a psychological condition; or (iv) residing outside the study catchment.

### Procedure

The frontline service providers were screened by service managers (or equivalent) within each of the four veterans’ support organisations. An informed consent form was provided to those who wished to participate. Those who attended the scheduled group session tendered their signed consent form, and completed a background questionnaire. The questionnaire took less than 5 min to complete. The facilitator distributed the discussion guide and the service providers were given an opportunity to independently work through the questions prior to the group discussion. The focus groups ranged in size from four to five participants and lasted 115 min (on average).

Veterans and family members who expressed an interest in participating in the study completed a brief screening interview over the phone. If eligible to participate, an informed consent form was sent via email. Prospective participants were contacted approximately 3 days later to enquire whether they had any questions, and whether they were interested in proceeding with the study. Those who wished to proceed were asked to email their signed consent form. The participant was emailed a background questionnaire and asked to return it prior to their scheduled interview. The questionnaire took approximately 10 min to complete. The one-on-one interviews lasted 55 min (on average). When a potential participant could not be reached at a given stage in the study procedure, up to two follow-up attempts were made before discontinuing contact. Written informed consent was obtained from all participants prior to data collection.

### Analysis

A reflexive thematic analysis [32, 33] of the focus group and interview transcripts was conducted using NVivo 12 Plus for Windows [34]. In the analysis, a theme is conceptualised as a pattern of shared meaning organised around a core idea (having both semantic and latent content), which is generated from smaller units of meaning (i.e., codes) [33]. An inductive approach was used to generate the preliminary codes and themes for the thematic analysis within a phenomenological framework [35]. After the initial inductive review of the data, the themes and codes were organised under the four study topics. Upon further review of the transcripts, additional codes were inductively added, and existing codes were inductively revised and reclassified under themes.

## Results

### Participation and response rates

All 18 service providers who expressed an interest in the study participated in the focus groups. Twenty-six veterans and family members who expressed an interest in the study were contacted regarding participation; 16 contributed interview data to the thematic analysis. Of the 26 individuals contacted: two could not be reached for screening; three were excluded because they were currently serving in the military; one was excluded because they resided outside the study catchment; one could not be reached for consent; two could not be reached for interview; and one was interviewed but the data was not transcribed as the content was not relevant to the focus of the study.

### Description of sample

All service providers who participated in the focus groups (*n* = 18) delivered services in metropolitan areas of South East Queensland; two providers also delivered telehealth services to veteran families residing in other states of Australia. Most service providers were female. The majority were employed full-time; the remainder worked part-time (up to 4 days a week). On average, service providers had 5.75 years’ experience working with veterans or their family members (range = 7 months to 40 years; median = 3 years) and had been in their current role for 2.75 years (range = 5 months to 15 years; median = 2 years). They were employed in a range of roles within veterans’ support services, and had a range of professional backgrounds. Most were providing mental health or advocacy services (with/without case management and referral) to veterans or their family members. Other types of service provision included: financial assistance; employment and training assistance; physical rehabilitation; and disability services. Two-thirds of the service providers had a lived experience of the military; having either served themselves or having one (or more) family members who served in the ADF (predominantly in the Army; the remainder split between the Navy and the Air Force).

All service users who contributed data to the qualitative analysis (*n* = 16) resided in South East Queensland. The majority of the sample were of Caucasian background, married, with more than one child. Seven of the service users interviewed were veterans (majority male with children over the age of 18); seven were female spouses of male veteran relatives (one widowed; all with children under the age of 18) and two were adult children of male veteran relatives (one male; one female). On average, veteran families had 12.94 years of lived experience of military service (range = 2 years to 40 years; median = 11 years) and had transitioned from full-time military service 17.19 years prior to participating in the study (range = 6 months to 48 years; median = 13 years). Over two-thirds of the service user sample had a history of operational deployment (i.e., combat, peacekeeping, and border protection duties in various locations including: Afghanistan; Iraq; Malaysia; Papua New Guinea; Singapore; Timor-Leste; Vietnam). Although not explicitly asked about number of deployments, multiple types and locations of deployment were more prevalent in the family member subsample, relative to the veteran subsample.

For the veteran subsample (*n* = 7): two reported a prior diagnosis of a psychological condition; three reported prior treatment for a psychological condition; and one reported a chronic mental health condition. Two veterans reported a history of serious injury; two reported a chronic pain condition (with impact on daily activities); and four reported a chronic physical health condition. Six of the seven veterans reported they had a service-related claim accepted; two reported holding an entitlement to fully-subsidized healthcare for all conditions; and four reported holding an entitlement to fully-subsidized healthcare for specified conditions. Six of the seven veterans reported engagement with two or more health and social services: three with mental health services; two with physical rehabilitation services; two with employment and training services; two with financial services; one with other health or social services; and seven with one or more veterans’ support organisations.

For the family member subsample (*n* = 9): three reported a prior diagnosis of a psychological condition; seven reported prior treatment for a psychological condition; and none reported a chronic mental health condition. Three family members reported a history of serious injury; one reported a chronic pain condition (with impact on daily activities); and four reported a chronic physical health condition. Five of the nine family members interviewed reported engagement with two or more health and social services: five with mental health services; one with employment and training services; one with financial services; one with advocacy services; and eight with one or more veterans’ support organisations. There is no data on service-related claims or entitlements for the family members interviewed as none were former-serving ADF personnel.

All family members (*n* = 9) reported that their male veteran relative (*n* = 9) had been previously diagnosed and treated for a psychological condition; all were reported to have a chronic mental health condition. Eight of the nine veteran relatives had reportedly sustained a serious injury; seven, a chronic pain condition (with impact on daily activities); and seven, a chronic physical health condition. All nine of the veteran relatives reportedly had a service-related claim accepted; seven were holding an entitlement to fully-subsidized healthcare for all conditions; and two were holding an entitlement to fully-subsidized healthcare for specified conditions. Two of the nine veteran relatives with an accepted service-related claim were pursuing additional claims: both had a claim in preparation and a claim outcome pending. Eight of the nine veteran relatives were reportedly engaged with two or more health and social services: eight with mental health services; five with physical rehabilitation services; one with employment and training services; four with financial services; six with advocacy services; one with housing services; two with other health or social services; and seven with one or more veterans’ support organisations.

There were two salient findings from the participants’ responses to background questionnaires: (i) the high proportion of frontline service providers with a lived experience of military service; and (ii) the preponderance of complex needs in the veteran families who chose to participate in the study. The questionnaire data provides an indication of the diversity of perspectives represented by the thematic analysis, and the extent to which the study findings may be transferrable to other settings. It also provides an indication of the success of the purposive sampling approach.

### Saturation assessment

The methodology developed by Guest et al. [36] was adapted to conduct a post hoc saturation assessment of the inductive thematic analysis (see Additional file 4). Using the four focus groups as the “base size” to calculate the “amount of information already gained”, and a “run length” of two interviews to calculate the “new information” gained; the analysis demonstrated that data saturation was achieved by the fourth focus group using a “new information threshold” of less than 5%, and by the fourth interview using a “new information threshold” of zero. Substituting a “run length” of three interviews (a more conservative approach), produced the same findings.

Braun and Clarke [37] have expressed criticism of saturation analysis in qualitative studies; particularly when the technique is employed in sample size estimation prior to, or in the early stages of, data collection. The saturation definition employed in this study accords with Malterud, Siersma, and Guassora’s [38] concept of “information power” as recommended by Braun and Clarke: that is, “the more relevant information a sample holds, the fewer participants are needed” [37]. The concept is perhaps best illustrated in this study by considering the high proportion of service providers with a lived experience of military service. This group of participants could draw on their experiences of the veterans’ support system, their experiences of military lifestyle and culture, and integrate these sources of knowledge; offering their service provider perspectives, the perspectives of the veteran families they support, and potentially their perspectives as service users (the background questionnaire did not explicitly ask service providers about their own use of support services). The richness of insight (i.e., information power) afforded by their multiple perspectives on the study topics may explain our data saturation findings. That is, if the sample had been comprised of service providers without a lived experience of military service, more interviews with veteran families may have been required to achieve data saturation within the sample.

### Thematic analysis

The findings from the reflexive thematic analysis [32, 33] are presented in Table 1. A summary of the themes is provided under each study topic, and anonymised participant comments are used to illustrate the findings.

**Table 1:** Overview of the study topics and themes from the reflexive thematic analysis

Health and wellbeing needs in veteran families	Service-access barriers and facilitators	Unmet needs and gaps in service provision	Practical solutions for improving service delivery
The veteran family experience Complex needs and caregiver burden	Service navigation Socioeconomic and military influences on help-seeking	Consistent service response Family-centred care Timely, holistic intervention	Discharge planning and continuity of care Features of an effective model of care

### Health and wellbeing needs in veteran families

#### The veteran family experience

Participants described how a lived experience of military service, and sociocultural factors, interacted to influence occupational and social opportunities, access to health and social services, and quality of life in veteran families. An understanding of the military lifestyle (e.g., trainings, postings, and deployments) and culture (e.g., sacrifice, stoicism, discipline, and teamwork) was considered essential for understanding health and wellbeing needs in veteran families. One service provider expressed this common participant view:

“What we do know is that children and families with a veteran have a very unique experience... when they're current serving and then when they transition out. But then there’s an expectation for that family to access mainstream services that aren’t actually equipped to understand that experience.” (Lived experience service provider)

Participants emphasised the benefits and challenges of military service for families. Whilst serving, veterans reportedly received comparatively high levels of remuneration, training-based opportunities for advancement, and priority access to health and social services. Membership of the military community was a source of pride, purpose, and meaning for veteran families; and provided a sociocultural context for the sacrifices they made in service of the nation. Nonetheless, over the longer term, military career demands were viewed as straining family functioning and adjustment:

“I think ultimately for everybody, for all veterans, at some point in your career it will come to a point where you decide between your family and your career.” (Lived experience service provider)

Concerns related to posting cycles focused on the cumulative effects of periodic relocation on partner employment (e.g., career prospects), children’s schooling (e.g., educational outcomes), and family support (e.g., continuity of childcare and healthcare; connection to extended family and friends). Training commitments and deployment cycles burdened family members, who had to repeatedly contend with the veteran’s absences (e.g., single parenting), and readapt as the veteran exited and re-entered the family system (e.g., family conflict).

Post-discharge, some veteran families reportedly struggled with civilian reintegration. Particular emphasis was given to veterans’ expectations of (and preparedness for) civilian employment environments, and the families’ disconnection (and sometimes exclusion) from the military community. One family who had experienced a medical discharge felt particularly aggrieved:

“Not one person from his unit has called him, from his discharge, in over a year. Yeah, not one person. And no-one’s reached out, nothing at all, yeah, complete radio silence, yeah … and it adds to the experience that he’s been through, you know, that he is unwanted, hey, that he is, you know, what do they say, like broken, thrown away, trash basically. Yeah, and I’ve been kicked out of some of the spouse groups on Facebook as well since discharge, yeah, which I find incredible as well. They’re not even Defence related.” (Family member)

Service providers shared their observations about a societal shift in veteran identity, and the role that the media played in instigating and perpetuating the narrative:

“You ask the average person on the street, ‘What do you know about veterans?’, and they’ll say, ‘They’ve all gone crazy. They’re all suicidal’. But then you ask them the next question, ‘Well, where do we have veterans deployed at the moment?’, ‘No idea’, because the good things that the military’s doing is just not reported, because it’s not newsworthy.” (Lived experience service provider)

#### Complex needs and caregiver burden

Service providers reported that complex needs were becoming increasingly common among the veteran families who were accessing their services and programs:

“I think what we’re seeing more and more is people coming in that are complex. They’re not usually coming in just with one thing going on. So, we’re seeing that more complex presentation more often. It’s not just one mental health (issue). A lot of the time, it’s housing, it’s financial; it’s lots of different things going on; relationship breakdowns.” (Service provider)

Those who were offering advocacy and referral support to veteran families affected by mental illness estimated that up to 100% were experiencing complex needs; of these, five to 30% were described as actively in crisis. Other service providers, whose roles gave them broad exposure to the veteran community, provided prevalence estimates for complex needs that were as low as 5%:

“This is not a reflection of the whole Defence Force. This is a very small portion of what’s happening, because there are a lot of people that have transitioned out of the Defence Force and their families… are quite well functioning, move through their lives, celebrate, have joy. Yeah, they endure difficulties, but they don’t ever come into contact with our system.” (Service provider)

The consensus view of participants was that civilian reintegration was a particularly vulnerable period for veteran families. The families that were perceived to be under the most pressure were those for whom the veteran had suffered service-related physical and psychological trauma, and for whom the sequelae of unresolved trauma had persisted over a prolonged period of time. Participants shared their concerns about the developmental and intergenerational effects of unresolved trauma, family system dysfunction, and family crises on minor and adult children:

“... it’s not just their PTSD, the fact that someone comes out unwell, but it’s the other effects of that, like the amount of domestic and family violence, the alcohol and drug misuse, the other criminal activity that happens because of all the bits and pieces, child safety issues, and just everything. It comes down to homelessness for some families… ” (Service provider)

An additional source of difficulty in families with an injured or ill veteran was the perceived impact of compensation and rehabilitation processes on veteran and family functioning. Service providers expressed concern that the burden of proof required to substantiate service-related claims was impeding rehabilitation efforts. The biomedical focus on pathology and diagnosis, and the medico-legal emphasis on deficits and incapacity, were perceived to harm veteran identity and undermine health and wellbeing outcomes.

Spouses of veterans with complex needs were reportedly experiencing very high levels of caregiver burden and significant impacts on their quality of life. They described performing multiple roles as carer, advocate, and case manager for their injured or ill veteran partner; in addition to their roles as a partner and parent; and (for those who were employed) their occupational role. The children in these families were coping with the relative incapacity of one parent while the other parent over-functioned to maintain the family unit.

“My kids have seen far too many things that I never wanted them to see, because I’ve had to learn how to manage his condition. I’ve had to learn what therapy looks like for him. I’ve had to become his psychologist. I’ve had to become his carer.” (Family member)

### Service-access barriers and facilitators

#### Service navigation

The consensus view of service providers was that veteran families had more services available to them than civilian families. However, a distinction was made between the availability of services, and providers’ and families’ capacity to navigate the crowded service system. Some family members who were supporting injured or ill veterans faced persistent difficulties locating services with an understanding of military lifestyle and culture, despite active help-seeking and relatively high levels of health literacy. For other veteran families, low levels of health literacy acted as a barrier to accessing services. Providers supporting these families leveraged their collective knowledge, skills, and experience to facilitate service access:

“Advocacy is actually such a huge part too in what we do because so many families we will go to visit, or we’ve spoken to, and they’ve said, ‘I’ve spoken to A, B, C and D but they won't listen.’” (Service provider)

“So, you have knowledge of how these services work, you know how to articulate the problems that the family is presenting with, and you can finesse that process?” (Interviewer)

“You know the language.” (Service provider)

Some service providers expressed concern about duplication of services, and the over-servicing of veteran families with complex needs. These issues were reportedly most prevalent when veterans with significant injury and illness were pursuing service-related compensation claims and entitlements. The consensus view of providers was that the demands of complex care coordination were not only overwhelming the capacities of families, but also of the professional supports who had been engaged to assist them with this process. For providers who were part of a team-care arrangement, some were experiencing difficulties tracking the number of other service providers involved, and following their (sometimes conflicting) treatment plans. One service provider expressed the phenomenon as follows:

“… it seems that a lot of my clients in particular have had far more service providers than say the general public would engage with, and multiple service providers at once. So, I have a client that is seeing our EP (Exercise Physiologist), seeing me (Psychologist), has a Psychiatrist, has a GP (General Practitioner), has a specialist for injury management; now has a speech pathologist… Has a Physiotherapist as well, or did? No. No, there’s some other specialist in there though... because there’s the skin specialist, the Dermatologist. Dermatologist, Endocrinologist. Oh geez… Sorry, there’s a social worker involved as well… Yep, social worker, and unfortunately the social worker can’t case manage it because there’s just too much.” (Service provider)

Case management and care coordination, advocacy and referral support, were unanimously viewed by participants as access facilitators. Flexible service delivery (e.g., telehealth) was viewed very favourably by families; some of whom were reportedly combining face-to-face psychological services with (crisis) phone counselling to manage periods of high psychological distress around planned appointments. Service providers valued discretion in the application of eligibility criteria, which allowed them to extend programs and services to families in need.

#### Socioeconomic and military influences on help-seeking

There were numerous barriers that reportedly affected veteran families’ capacities to access health and social services across multiple sectors (e.g., full-time work commitments, geographical distance, practitioners not accepting new clients, long wait lists and high caseloads, financial costs or funding limitations, and inaccessibility of childcare). Lack of social support, and prior (negative) experiences of services, acted as persistent, generalised barriers to help-seeking.

Military values and beliefs pervaded cultural norms in veteran families within and across generations. Two of the most prevalent factors that contributed to significant (and sometimes prolonged) delays in help-seeking were stoicism and stigma. In respect of stoicism, both family members and veterans appeared to have endured prolonged pain and hardship without complaint or display of emotion. This could be a cultural feature of veteran families that may place them at higher risk when experiencing complex needs. In respect of stigma, there were (rational) concerns that help-seeking could jeopardise career prospects or current employment (e.g., for veterans planning to, or working in, first responder networks post-discharge).

Other barriers were related to readiness to access services, with veterans sometimes positioned as information gatekeepers (e.g., family members requiring details about the veteran’s military service to meet eligibility criteria; veterans not sharing information about their health and wellbeing status with family members; veterans not advising family members about available services and supports). Some service providers perceived the persistence of military culture as an impediment to successful civilian reintegration:

“‘What if I say you’re not in the military anymore? What does that mean to you?’ They’re just like, ‘Once a solider, always a soldier’. I was like, ‘You’re talking about five years, six years, 10 years, 20 years of your life. You’ve got 40, 60 years left. What now? What do you want to do?’.” (Lived experience service provider)

A particular emphasis was placed on the importance of military-informed service provision as a significant access facilitator: it was unanimously viewed by participants as a factor that promoted service engagement and treatment retention.

### Unmet needs and gaps in service provision

#### Consistent service response

Some service providers identified a need for greater regulation of veterans’ support services to facilitate consistent professional standards. Participants expressed concern about: (i) variability in families’ experiences of service provider interactions over multiple contacts with the same agency; and (ii) variability in eligibility criteria for programs and services across different agencies.

“... they’re already so exhausted, sick of telling their story, sick of just this constant level of rejection through the system, for whatever reason, because there’s so many loopholes and they don’t meet this criteria or that one. And it really just depends on who you speak to on the day as to what response you get, and if you’re eligible or not, and the language that you use. So then when they come here, they’re like, ‘We need help,’ but they can’t even articulate what they want.” (Service provider)

Many of the veteran families who participated in the study were not aware of recent changes to eligibility criteria (e.g., broadening the definition of family member; extending services to family members that were previously only offered to veterans). In fact, three of the family members interviewed were provided with referrals to services on this basis.

#### Family-centred care

The experience of participants was that veterans’ support services could do more to involve family members in the care of veterans who were injured or ill:

“And, I think that there are a lot of services out there, but they’ve been veteran-centric. It has been around the veteran. It’s been very – the family has been a kind of spare wheel. They go along for the ride. They’ve been to the side, and it’s always been the veteran in the middle. And, I think it needs to be veteran and family in the middle, and you address it in that way.” (Service provider)

Legislative restrictions, privacy and confidentiality provisions, and veteran-centric organisational culture and eligibility criteria, were reported to impact the process of involving families in care coordination.

For the family members interviewed, the most consistent unmet need, which created a significant barrier to accessing services, was the inaccessibility of flexible (i.e., casual or respite) childcare:

“my big point is, why we need crèche (childcare), is because, number one, we need to value couples, but also we’re all Defence families, we all have no (local) family, friends and connections, I mean, all around Australia. So that’s my really big push, you know, for any organisation.” (Family member)

What is unfortunate about the unmet need for childcare was that it propagated further unmet need: services had reportedly rationalised family-centred [39] program offerings (e.g., psychoeducation on PTSD for carers and families) due to a perceived lack of demand (i.e., insufficient expressions of interest). Other highly-valued, family-centred programs had been scaled back due to funding shortfalls (e.g., weekend camps for children living in families affected by mental illness).

Participants identified a range of other areas where there were gaps in military-informed service provision; including: training to assist practitioners to differentiate neurodevelopmental disorders (e.g., attention deficit hyperactivity disorder, autism spectrum disorder) from developmentally-appropriate responses to military transitions (e.g., child internalising and externalising behaviours related to training, posting, and deployment cycles, and service-related injury, illness, and bereavement); military-informed parenting interventions; interpersonal (e.g., assertiveness) skills training for adult children and spouses of veterans; interventions to address conflict and violence in veteran families (including advocacy support for elder abuse); and family-centred social connection opportunities to address isolation from extended family and friends, and disconnection/exclusion from the military community.

#### Timely, holistic intervention

Service providers unanimously agreed that the transition seminars offered to veterans discharging from full-time service were not effectively preparing them for civilian reintegration. Timely, holistic intervention was identified as a primary unmet need.

“I found all the organisations very pigeonholed, if that's the right word, because they only look after their sort of one little thing. So, if I rang one organisation they'd say, ‘We can help you with this and this’, but then I'd have to come up with my own idea to ring another one… on the off chance that they might be able to help me. There's no interconnection between sort of saying, ‘Oh well, if you're a person and you're ringing up and asking if there's X, Y, Z, then you might also want to ring [this organisation], or someone else, or someone else, because they might also be able to help you’. There was no closing of the circle. They were just dealing with my specific request and I might not necessarily have been asking the right questions.” (Family member)

Families with complex needs often gained access to integrated care [40, 41] at the point of crisis (sometimes in relation to a veteran suicide attempt) after prolonged periods of psychological distress, and progressive deterioration in veteran and family functioning. These families were reportedly very grateful for the assistance they received, but lamented the delay in access:

“The one thing I do want to say is that I'm very grateful for all the help that we have been provided so far. I just wish it didn't take as much effort or as many questions or phone calls to get it all in place.” (Family member)

### Practical solutions for improving service delivery

#### Discharge planning and continuity of care

There was a consensus view among participants that better discharge planning, which more fully involved carers and families, could improve transition pathways from military to civilian environments:

“I think especially when people are transitioning out, it’s that access to information, and being aware of services and supports, and having those plans, having those discussions before that point; especially if people are medically discharging, or it’s happening quite quickly. I think that’s one of the barriers to how then people go onto access, or not access, these services for a long time, is that planning through transition, and families and primary caregivers being a part (of that).” (Service provider)

Service providers emphasised the challenges veterans faced when trying to find meaningful work in civilian environments. First responder roles (e.g., police, fire, ambulance) were perceived to offer features of military roles that were highly valued by veterans. Nonetheless, for some families these work environments offered further occupational exposure to trauma. A particularly salient example of an effective solution for managing the veteran transition to civilian employment was provided by the following service provider:

“... the people that I’ve worked with over the years who have been the most successful with their transitions have been people that have, while in Defence, taken unpaid leave and have gone and worked in the civilian workforce voluntarily. Because they’ve got that work, they’ve got connections, they know people, they can set up a LinkedIn profile, (and) they can keep in touch with people… they’ve got some roots outside of Defence.” (Service provider)

Participants felt that a number of innovations could improve providers’ and families’ awareness of existing services, and their capacity to navigate the crowded service system. There were a series of suggestions that involved leveraging or improving existing Information and Communication Technology (ICT) platforms; including: improvements to the usability and quality of various online service portals; changes to client management systems to better support service provider communication (i.e., case management and review, care coordination, and referral processes within and between services); national legislative reform and improved information sharing between Defence, DVA, and the veterans’ non-profit sector to reduce the burden of compensation and rehabilitation processes; family assessments at the point of discharge from full-time service; and assertive outreach (up to 18 months post-discharge) from Defence to veteran families to facilitate needs-based access to services.

#### Features of an effective model of care

There was a consensus view that training was required to ensure service provision was informed by an understanding of military lifestyle and culture. Participants highlighted the importance of funding incentives in developing family-centred models of care (e.g., flexible funding arrangements for crisis support). One family member shared a practical solution for childcare:

“… crèches (run) through… medical centres. There’s actually no qualifications required. All you need is a safe space, activities, and someone to supervise the children who has the clearances.” (Family member)

Participants emphasised the value of recovery-oriented practice [42] frameworks for injured or ill veterans who were pursuing service-related compensation claims and entitlements. The following service provider interaction illustrates their suggested changes to language and messaging to more strongly connect care to recovery:

“… move away from labels such as… ‘TPI’ (Totally and Permanently Incapacitated)... towards more like, ‘I’m rehabbing’ or ‘I’m transitioning’. So, a change in the language that we use with veterans to kind of encourage growth and recovery...” (Service provider)

“So, ‘We’re not saying you have to go to work this week… but we’re transitioning you from what you did in Defence to now being in a civilian capacity, and what we’re going to try and help you achieve in your new life away from Defence’. Rather than saying, ‘Okay, you’re totally and permanently incapacitated and that’s it for you, and that’s because of your military history. You’re now finished for life’.” (Another service provider)

Participants offered many practical solutions to address specific challenges faced by the veterans’ support system, but they seemed to be in resounding agreement on one thing: an integrated, holistic model of care [43] is required to improve health and wellbeing outcomes for veteran families. One family member’s vision for the veterans’ support system represents this common participant view of a decentralised, “no wrong door” approach to service delivery:

“... if everything was integrated, then hopefully one of my phone calls and one of my questions would have triggered something, which then would have started the whole process. Hopefully, in the future, it will be a lot easier if that happens.” (Family member)

## Discussion

The purpose of the study was to gain an in-depth understanding of complex needs in veteran families; and explore service providers’ and families’ experiences of the veterans’ support system. Both providers and families found it difficult to access and navigate the many services offered by military and civilian systems of care. System fragmentation was perceived to impede care coordination, and delay access to holistic care for veteran families with complex needs. The medico-legal aspects of compensation and rehabilitation processes were perceived to harm veteran identity, and undermine health and wellbeing outcomes. Recovery-oriented practice was viewed as a way to promote veteran independence and self-management. Participants expressed a strong preference for family-centred care that was informed by an understanding of military lifestyle and culture.

The findings from this study confirm and extend the findings from Muir’s [25] qualitative study of family members’ experiences of civilian reintegration. Muir’s study oversampled veteran families with a relatively high risk for a “challenging” reintegration experience (i.e., families where the veteran had experienced medical discharge and/or mental health conditions). He focused on four study topics: (i) family perceptions of “successful” reintegration; (ii) factors associated with better reintegration outcomes for veterans; (iii) the family’s role in “successful” reintegration; and (iv) strategies for improving family reintegration outcomes.

Consistent with the findings from this study, the veteran families in Muir’s study [25] were profoundly affected by the transition from full-time military service to civilian environments. Families’ perceptions of successful reintegration were heavily influenced by their specific circumstances. The families most negatively affected by civilian reintegration were those in which the veteran struggled with serious health conditions and/or with difficulties finding civilian employment. Veterans with complex needs required extensive family support. Compensation claims processes were perceived to exacerbate existing mental health conditions. Families’ variable knowledge of the veterans’ support system, and military influences on help-seeking (e.g., stoicism and stigma), acted as significant barriers to accessing and engaging appropriate services. Better reintegration outcomes were reported when families were involved in timely discharge planning, and formal and informal supports had an understanding of military lifestyle and culture. Improved communication with families, and family-centred case management and care coordination, were viewed as essential for improving family reintegration outcomes.

The findings from this study extend those of Muir [25] by including service providers’ perspectives on veteran families with complex needs, and providers’ experiences of accessing and navigating the Australian veterans’ support system. Critically, this study provides important insights into precisely which aspects of the system would benefit from efforts to better integrate and coordinate care for vulnerable families. The identified access barriers and facilitators, unmet needs, and gaps in service provision provide specific targets for improvement activities. Many of these activities require collaboration between services. However, the literature does not offer a readymade solution: no high-quality implementation studies are available to design an optimal service response for veteran families with complex needs [44].

In Australia, one option that bears consideration is the Partners in Recovery (PIR) initiative. PIR was specifically designed to improve health and wellbeing outcomes for consumers with severe, persistent mental illness, and complex needs, by improving intersectoral care coordination. The target population was known to have relatively high levels of unmet need, a requirement for more intensive support to address the complexity of their needs, and a tendency to “fall through the gaps” in fragmented systems of care [45]. The sectors that were considered fundamental to the initiative included: primary, secondary, and tertiary healthcare; state and territory mental health systems; the non-government health and social services sector; alcohol and drug services; disability services; justice and safety services; income support services; and education, employment, and housing services [45]. The ultimate aim of the PIR initiative was to ensure that consumers with complex needs were accessing timely, holistic, effective, integrated care.

The PIR model of care was guided by a series of core principles: it was family-centred and recovery-oriented; it was designed to be flexibly tailored to local service networks and the needs of local consumers; it was intended to complement (rather than supplant or duplicate) existing service systems; and it focused on simplifying service navigation by building networks and referral pathways between sectors, services, and supports [45]. Certain features of the PIR model of care were considered to be particularly innovative with respect to contemporary practice at the time. Namely, the consortium model of interagency collaboration, the structure of the care coordinator role, and the provision of a (limited) flexible funding pool to commission services to address gaps in the service system [45]. The model incorporates many of the strategies employed to address fragmented systems of care (for systematic reviews see Whiteford et al. [46] and Thomas et al. [47]). Furthermore, although not explicitly identified by participants in this study as a potential system integration solution, the PIR model of care is strongly aligned with many of their recommendations.

### Limitations

The study recruited service providers and veteran families who were working and living in metropolitan areas of South East Queensland, respectively: the participants’ perceptions may be limited by their experiences of services within this region. The veteran families who participated in the study were predominantly traditional and heteronormative in structure; comprising a male veteran, female civilian spouse, and one or more children [4]. In future research, it may be useful to explore the experiences and perspectives of non-traditional families [4], and family members having other types of relationships with their veteran relative (e.g., parents, grandparents, siblings). This work could potentially reveal additional service-access barriers and facilitators, unmet needs, gaps in service provision, and strategies for improving system integration across the transition pathways from military to civilian environments.

## Conclusions and future research

The health and wellbeing needs of veteran families intensify during the transition from full-time military service to civilian environments, and service- or reintegration-related difficulties may emerge (or persist) for a significant period of time thereafter (see also [17]). Veteran families with complex needs are unduly burdened by care coordination demands. There is an urgent need for high-quality implementation studies that evaluate initiatives for integrating fragmented systems of care (see also [44]). The lack of evidence-based options presents a significant barrier to policy development, strategic service planning, and resource allocation across the veterans’ support system.

The PIR model of care was implemented by 48 regional consortia across 61 service regions in Australia: it is the only largescale, integrated care initiative for people with complex needs that has been implemented nationally. Although there is no definitive evidence that PIR “worked”, there is much that the veterans’ support system can learn from the published literature on the implementation challenges faced by these various service networks (see Additional file 5). As randomised controlled trials of these types of initiatives are likely to be infeasible, it is important that implementation efforts are supported by sophisticated research strategies that provide robust evidence of their success or failure (e.g., see [48]). These efforts will require continued feedback from service providers and veteran families using co-design [26] approaches similar to that employed in this study. Implementation research is resource-intensive and funding is scarce. Further investment from governments and funding bodies is required to incentivize strategic partnerships between researchers, services and families to develop evidence-based solutions that are sustainable in real-world settings [26].

## Supplementary Information

**Additional file 1.** Key terms, concepts, and operational definitions

**Additional file 2.** Focus group protocol – service providers

**Additional file 3.** Interview protocol – veteran families

**Additional file 4.** Saturation assessment of inductive thematic analysis

**Additional file 5.** Partners in Recovery peer-reviewed literature

## Data Availability

The datasets generated and analysed during the study may be made available upon reasonable request to the corresponding author subject to the written approval of the GMRF Research Advisory Committee and the DDVA HREC.

## Abbreviations

ADF: Australian Defence Force
CSC: Commonwealth Superannuation Scheme
COREQ: COnsolidated criteria for REporting Qualitative research
DMFS: Defence Member and Family Support
Defence: Department of Defence
DVA: Department of Veterans’ Affairs
DDVA HREC: Departments of Defence and Veterans’ Affairs Human Research Ethics Committee
ESO: Ex-Service Organisation
GMRF: Gallipoli Medical Research Foundation
HHSs: Hospital and Health Services
ICT: Information and Communication Technology
MBS: Medicare Benefits Scheme
NDIS: National Disability Insurance Scheme
PIR: Partners in Recovery
PBS: Pharmaceutical Benefits Scheme
PTSD: Post-traumatic Stress Disorder
PHNs: Primary Health Networks
RPBS: Repatriation Pharmaceutical Benefits Scheme
RSL Queensland: Returned & Services League of Australia, Queensland Branch
TPI: Totally and Permanently Incapacitated

## Footnotes

1 Of the total cohort (*n* = 24,932); 96.2% (23,974) were invited to participate (i.e., did not opt out of the study; had usable contact information) and 18% (*n* = 4326) completed the survey [17]. Of those that completed the survey, 41% (*n* = 1807) were selected for a Composite International Diagnostic Interview and 24% (*n* = 1049) completed the interview [17]. To generate estimates for the entire population, the data was weighted using distinct strata for sex, rank, medical fitness, and Service branch (i.e., Army; Navy; Air Force) [17].
