# Supplementary material for: Veteran families with complex needs: a qualitative study of the veterans’ support system
Source: BMC Health Serv Res. 2022 Jan 15;22:74. doi: 10.1186/s12913-021-07368-2 (PMC8760758; doi:10.1186/s12913-021-07368-2)
Supplement: Supplementary file 1 — Additional file 1. Key terms, concepts, and operational definitions [file 12913_2021_7368_MOESM1_ESM.pdf]

Additional file 1. Key terms, concepts, and operational definitions

Term/Concept	Operational Definition
Ex-service organisation(s), ESO(s)	An Australian not-for-profit charity or trust providing health and/or social services to veterans and/or their family members.
Family-centred care	A model for delivering health and social care that emphasises partnership between service providers, service users, and their carers and families [39]. Family-centred care is intended to empower individuals and families by engaging them in shared decision-making regarding their health and social care [39].
Family member(s)	A spouse, partner, child, parent, step-parent, step-child, adopted child, grandparent, grandchild, sibling, in-law, and persons who 'stand in the position' of a parent or child. The definition of family member employed for the study aligns with the definition of "dependant" under the Military Rehabilitation and Compensation Act (MRCA), and was purposefully chosen to reflect common family structures in Australian society.
Frontline service provider(s)	A paid employee or volunteer providing frontline health and/or social services to veterans and/or their family members.
Integrated care	Care that is based on the needs of individuals and/or populations, which is coordinated across service providers, service organisations (e.g., clinical and non-clinical services), and service systems/sectors (e.g., primary, secondary, and tertiary/quaternary care) to achieve "triple aim" outcomes: improved care experience (for both service users and service providers), improved population health, and reduced per capita care cost [40]. Note that integrated care may uncover unmet need: addressing unmet need may improve health outcomes for individuals and/or populations, but per capita care cost is likely to rise [41]. Even so, integrated care may still offer value for money if it improves quality of life [41].
Lived experience	Lived experience refers to an individual's experiences and choices, and the knowledge and skills they gain from these experiences and choices [1].
Metropolitan areas of South East Queensland, Australia	South East Queensland is a bio-geographical, political, and administrative region of the state of Queensland, Australia, which is bounded by Noosa in the North, Tweed Heads in the South, Toowoomba in the West, and Brisbane in the East. Metropolitan areas include the following five regions of South East Queensland: the City of Brisbane (the capital city of Queensland and largest Local Government Area in Australia); Ipswich City (an outer-suburban city with an industrial and mining heritage west of Brisbane); Logan City (a largely residential area between Brisbane and the Gold Coast); the Moreton Bay Region (a largely residential area between Brisbane and the Sunshine Coast); and Redland City (a residential and agricultural area on the shores of Moreton Bay to the south-east of Brisbane City).
Military-informed	An understanding of military lifestyle (e.g., training, posting, deployment cycles) and culture (e.g., language, communication styles, values and beliefs, attitudes and norms).
Model of care	A model of care broadly defines the way in which health and social services are delivered; outlining the manner in which an individual, group, or target population will be supported through the stages of an injury, condition, or event [43].

Posting cycle(s)	A posting cycle is a process whereby military personnel are appointed to a new positions or roles (including promotions) within the Defence force [14]. Postings may require geographical relocation from one military base to another (i.e., from the 'losing location' to the 'gaining location') [14]. In the Australian Defence Force (ADF), postings occur on an annual basis, usually taking effect from December to January [14]. Typically, ADF personnel will be posted for duty to a different position, role, or military base every two to three years.
Recovery-oriented practice	Recovery-oriented practice focuses on engendering hope, building resilience, and promoting self-determination; and is often viewed as an alternative to the "medical model" emphasis on diagnosing and curing injury and illness [42]. Within this framework, "clinical recovery" (i.e., alleviation of symptoms or cure) is typically contrasted with "personal recovery" (i.e., creating a meaningful and contributing life regardless of health status) [42]. Recent work has emphasised the relationship between clinical recovery and personal recovery, viewing these states as complements rather than alternatives [42].
Veteran(s)	A former serving member of the military having had one or more days of continuous full-time service in the Permanent Forces (i.e., not the Reserve Forces). Note that a broader definition of "veteran" includes current-serving military personnel in both the Permanent and Reserve Forces. Due to recruitment considerations, the former definition was used for the qualitative study, and the latter definition is used throughout the remainder of the paper.
Veteran families	Families with a lived experience of military service (i.e., either as veteran, or as the family member of a veteran).
Veterans' support organisations	Government and non-government organisations (NGO) and agencies, ex-service organisations (ESOs) and not-for-profit charities or trusts, providing health and/or social services to veterans and/or their family members.
