# Supplementary material for: Veteran families with complex needs: a qualitative study of the veterans’ support system
Source: BMC Health Serv Res. 2022 Jan 15;22:74. doi: 10.1186/s12913-021-07368-2 (PMC8760758; doi:10.1186/s12913-021-07368-2)
Supplement: Supplementary file 2 — Additional file 2. Focus group protocol – service providers [file 12913_2021_7368_MOESM2_ESM.pdf]

Additional file 2. Focus group protocol – service providers

Introductions: facilitator(s), GMRF, and project

Hi everyone, my name is [name]. I'm a [role and qualifications] from Gallipoli Medical Research Foundation (GMRF). GMRF is a not-for-profit, approved research institute based in Brisbane, Australia. We conduct health research with current serving and ex-serving ADF personnel and their families on topics that are relevant to their physical health, mental health, and social wellbeing.

The focus group that you have agreed to participate in today is part of an effort to better understand the needs of families with a veteran. As part of this study, we're asking frontline service providers about the health and wellbeing needs of families with a veteran. We are interested in understanding how well those needs are being met by support services. We're also talking to service users about their needs and their experiences of support services.

We're hoping to talk to people with different professional backgrounds and different experiences of supporting veterans and/or their family members. This will help us to understand the range of problems that veterans and their families are dealing with and the types of support needs they have. It will also help us to identify whether there are any gaps in the services that are offered to veterans and their families, and whether there is anything we can do to improve service delivery.

GMRF will use the information you share with us today to develop a set of recommendations that support services could use to coordinate the programs they deliver to veterans and their families. This could include recommendations about how to measure the needs of veteran families, how well services are doing in terms of meeting those needs, and opportunities to improve services.

Duty of care, consent, privacy and confidentiality

Although we're recording this session today, we won't disclose your personal information to anyone outside the project. We will keep the information that you share with us confidential. However, if you tell me that someone is being harmed, especially if a child or an elderly person is being harmed, you should know that I may be required to report the harm to the appropriate authorities.

GMRF will destroy the questionnaires, focus group recordings and notes, and transcripts of the focus groups at the end of the minimum period, which is 5 years from the date when the de-identified findings are reported. In the meantime, the information will be held in a secure area of GMRF on a password-protected computer.

Taking part in this focus group is voluntary—you don't have to answer any questions that you don't want to answer, and you don't have to discuss any topic that makes you feel uncomfortable. I appreciate any insights you can share with us; but please let me know, at any time, if you don't want to participate or if you want to stop participating. I would encourage you to respect each other's privacy by not revealing names and comments to people outside the focus group, but I cannot guarantee or promise that people will follow that guidance.

Check understanding

Does anyone have any questions before we get started?

Discussion guide

So, I've created a guide for the focus group to help us structure the group discussion we are going to have [distribute form].

**PRESENTING PROBLEMS**	
Service User (Box 1)	Family (Box 2)
**SUPPORT YOU PROVIDED**	
Service User (Box 3)	Family (Box 4)
**INTERNAL REFERRALS**	**EXTERNAL REFERRALS**
Who and how (Box 5)	Who and how (Box 9)
What went well (Box 6)	What went well (Box 10)
What didn’t go well (Box 7)	What didn’t go well (Box 11)
What you would change about the process (Box 8)	What you would change about the process (Box 12)
**ACCESS TO PROGRAMS & SERVICES**	
Barriers (Box 13)	Facilitators (Box 14)
**TOP 3 CHANGES YOU WOULD MAKE TO THE SERVICE SYSTEM (HIGHEST TO LOWEST IMPACT)**<br>1.<br>2.<br>3.	
**OTHER COMMENTS:**	

Context

As I said earlier, the purpose of the research is to develop a set of recommendations to help guide service delivery for families with a veteran. We are holding focus groups with frontline service providers because we want to give you a voice in this process. This is important research, and we are really interested in your experiences and opinions. We really value the time you are giving to the focus group today. I am here today as a facilitator, not as a participant. I have put together this form as a way of guiding your discussion about the support needs of families with a veteran.

Process

I thought we could start today by giving you some time to reflect on your experiences of supporting service users and their families. You will then have an opportunity to consider each other's experiences by sharing these forms around the room. Then we will have a break and when we come back, I would like to facilitate a group discussion about your responses to the form. At the end, there will be time for you to share anything that hasn't already been covered through this more guided discussion process. Does anyone have any questions?

Introductions: group members

Ok so let's start by introducing ourselves: the focus group responses are confidential but the person transcribing the audio-recording will need to hear your name, your service provider role, and your organisation in your OWN VOICE to assign you an ANONYMOUS participant number. This is so we can analyse all the responses we are collecting as part of this study.

So, I'll start… My name is [name]; I am a [role and qualifications]; and I work for Gallipoli Medical Research Foundation.

Can I ask you to go next? Say your name, your current role, and your organisation and then we will go around the room…

Problems

I'd like to give you some time now to think about a service user who you have provided support to in the last 6 months. I want you to focus on someone who had problems that you found difficult to address in the current service system.

1. In Box 1, list the problems the service user was having…

[prompt for physical health; mental health; social wellbeing]

2. Now I want you to think about any problems that you were aware of in their family system; list those problems in Box 2… if you don't have this information; just write "I don't know" in Box 2…

[prompt for physical health; mental health; social wellbeing]

Support you provided

Now let's think about the support you provided. Look at the service user and family problems that you listed in Box 1 & 2. In your role as a service provider, list the support you provided to the service user in Box 3 and to their family in Box 4. If you didn't provide any support to the family, just leave that box blank.

Internal referrals

Now let's think about internal referral pathways. Which service user or family problems were you able to address by referring them to another service provider in your own organisation? In Box 5, list who you referred them to and how you referred them (e.g., form; phone call; face-to-face)? In Box 6, list anything that went well with the internal referral process. In Box 7, list anything that didn't go well. In Box 8, list anything you would change about the internal referral process to improve it.

External referrals

Now let's think about external referral pathways. Which service user or family problems were you able to address by referring them to another service provider that was outside your organisation? In Box 9, list who you referred them to and how you referred them (e.g., form; phone call; face-to-face)? In Box 10, list anything that went well with the external referral process. In Box 11, list anything that didn't go well. In Box 12, list anything you would change about the external referral process to improve it.

Barriers

Now I want you think about whether there were any gaps in the service network (i.e., unmet service user needs or family's needs) where the service wasn't available for some reason (e.g., not offered; no funding; long wait lists; service doesn't exist)? In Box 13, list the problems you faced in trying to get this service user or their family the support they needed…

Facilitators

In Box 14, list anything that helped when you were trying to get the service user or their family the support they needed.

Top three changes

Now consider everything we have worked through today. If there were three things you could change about the current service network; what would they be? Please list the changes in order of those things that you think would have the highest to lowest impact on the service users and families you are supporting…

Other comments

I've just left a space at the bottom for you to make any additional notes because we are going to have a group discussion after the break and I just want you to put some notes in there for yourself about points you might want to raise later about anything we haven't covered as part of this structured process that we should be having a chat about in relation to providing support to veterans and their families. Things that would assist you as a service provider or things that would assist the families or both. Any comments you have, any experiences that you have that you would like to share… that you think would be helpful in terms of developing recommendations for how we should be delivering services to veterans and their family members.

Review colleagues' responses

Now I would like to give you an opportunity to share your responses around the table… so if we can rotate the forms that you've been writing on. This is just so you have an opportunity to review each other's responses. Once that's done we'll have a break for 10 minutes and we can come back and have a group discussion about your responses to the discussion guide we have been working through today…

Break

What I'd like to do now is give you a break… to grab tea / coffee / biscuits and then we'll come back in 10 minutes and we'll work through the form together and have a discussion about what came up for you in each section… things that you noticed were common experiences, things that were unique and we'll take it from there…

Group discussion

Now I would like to bring you all the way back round to the beginning again to give you an opportunity to share your experiences. I'm really interested in what you have to say about what we've worked through and what you have to say about reviewing each other's responses. So if we can start with presenting problems… who would like to go first?

Concluding remarks

Thank you for being so generous with your time and for sharing your experiences and insights. Your contribution today will help us to better understand some of the challenges that veterans and their family members are facing in terms of managing their health and wellbeing needs. It will also help us to identify whether there are any gaps in the programs that are being offered to veterans and their family members.

Before we wrap up, are there any questions that you have, or any last comments you would like to make? If you have questions about the study that you think of later, my contact information is on the participant information sheet and consent form. Thanks again for coming along today…
