# Supplementary material for: Veteran families with complex needs: a qualitative study of the veterans’ support system
Source: BMC Health Serv Res. 2022 Jan 15;22:74. doi: 10.1186/s12913-021-07368-2 (PMC8760758; doi:10.1186/s12913-021-07368-2)
Supplement: Supplementary file 3 — Additional file 3. Interview protocol – veteran families [file 12913_2021_7368_MOESM3_ESM.pdf]

Additional file 3. Interview protocol – veteran families

Introductions: facilitator(s), GMRF, and project

Hi [name], my name is [name]. I'm a [role and qualifications] from Gallipoli Medical Research Foundation (GMRF). GMRF is a not-for-profit, approved research institute based in Brisbane, Australia. We conduct health research with current serving and ex-serving ADF personnel and their families on topics that are relevant to their physical health, mental health, and social wellbeing.

The interview that you have agreed to participate in today is part of an effort to better understand the needs of families with a veteran. As part of this study, we're talking to service users about their needs and their experiences of support services. We are interested in understanding how well those needs are being met by support services. We're also talking to frontline service providers about the health and wellbeing needs of families with a veteran.

We're hoping to talk to people with different family backgrounds and different experiences of support services. This will help us to understand the range of problems that veterans and their families are dealing with and the types of support needs they have. It will also help us to identify whether there are any gaps in the services that are offered to veterans and their families, and whether there is anything we can do to improve service delivery.

GMRF will use the information you share with us today to develop a set of recommendations that support services could use to coordinate the programs they deliver to veterans and their families. This could include recommendations about how to measure the needs of veteran families, how well services are doing in terms of meeting those needs, and opportunities to improve services.

Duty of care, consent, privacy and confidentiality

Although we're recording this session today, we won't disclose your personal information to anyone outside the project. We will keep the information that you share with us confidential. However, if you tell me that someone is being harmed, especially if a child or an elderly person is being harmed, you should know that I may be required to report the harm to the appropriate authorities.

GMRF will destroy the questionnaires, interview recordings and notes, and transcripts of the interviews at the end of the minimum period, which is 5 years from the date when the de-identified findings are reported. In the meantime, the information will be held in a secure area of GMRF on a password-protected computer.

Taking part in this interview is voluntary—you don't have to answer any questions that you don't want to answer, and you don't have to discuss any topic that makes you feel uncomfortable. I appreciate any insights you can share with me; but please let me know, at any time, if you don't want to participate or if you want to stop participating.

Check understanding

Do you have any questions before we get started?

Context

As I said earlier, the purpose of the research is to develop a set of recommendations to help guide service delivery for families with a veteran. We are holding interviews with service users because want to give you a voice in this process. This is important research, and we are really interested in your experiences and opinions. We really value the time you are giving to the interview today.

Process

I thought we could start today by giving you some time to reflect on your experiences, and your family's experiences, of veteran's support services.

1. Since the ADF discharge process started, have you faced any challenges in terms of getting your health and wellbeing needs met? What about the needs of your family members?

   [Prompt for service-access facilitators and barriers related to: services (e.g., financial/ geographical), service providers (e.g., role/lived experience) and service users (e.g., individual/family system/military or veteran culture).]

2. Do you have any preferences (e.g., age, gender, professional background, lived experience) in relation to who you feel most comfortable/confident seeing for support?

   [Prompt for factors (e.g., individual / family system / military or veteran culture) that may influence the needs, values and preferences of the service user in relation to health and social care. These factors could impact an individual's help-seeking behaviours, their propensity to access and/or stay engaged with services, and their perceptions of services.]

3. How do you view the health and social support services with whom you DO engage?

   [Prompt for the availability of programs for veterans or veteran families with high levels of need.]

4. How do you view the health and social support services with whom you DON'T engage?

   [Prompt for the availability of programs for veterans or veteran families with high levels of need.]

5. Do you have any preferences in relation to who manages your care (e.g., GP)?

   [Prompt for preferences in relation to point-of-entry to services network (i.e., centralized or de-centralized access). Prompt for preferences in relation to which provider(s) should have responsibility for care coordination once the service user has engaged with services.]

6. Are there things you think the veterans' services network does well?
7. Are there things you think the veterans' services network could do better?
8. If you could change one thing about the veterans' services network, what would it be?
9. Overall, what is your general attitude towards the health and social services that provide support to veteran families?
10. Overall, how would you describe your general experiences with the health and social services that provide support to veteran families?

Concluding remarks

Thank you for being so generous with your time and for sharing your experiences and insights. Your contribution today will help us to better understand some of the challenges that veterans and their family members are facing in terms of managing their health and wellbeing needs. It will also help us to identify whether there are any gaps in the programs that are being offered to veterans and their family members.

Before we wrap up, are there any questions that you have, or any last comments you would like to make? If you have questions about the study that you think of later, my contact information is on the participant information sheet and consent form. Thanks again for coming along today…
