# Supplementary material for: Veteran families with complex needs: a qualitative study of the veterans’ support system
Source: BMC Health Serv Res. 2022 Jan 15;22:74. doi: 10.1186/s12913-021-07368-2 (PMC8760758; doi:10.1186/s12913-021-07368-2)
Supplement: Supplementary file 4 — Additional file 4. Saturation assessment of inductive thematic analysis [file 12913_2021_7368_MOESM4_ESM.pdf]

Additional file 4. Saturation assessment of inductive thematic analysis

	**Data Collection Event**																			
	FG1	FG2	FG3	FG4	IN1	IN2	IN3	IN4	IN5	IN6	IN7	IN8	IN9	IN10	IN11	IN12	IN13	IN14	IN15	IN16
**New Codes**	39	6	12	5	0	2	0	1	0	0	0	0	0	0	0	0	0	0	0	0
**Base Codes**				**62**																
**Run length = 2**																				
**New Codes in Run**						2	2	1	1	0	0	0	0	0	0	0	0	0	0	0
**Saturation Ratio* (%)**						**3.23**	3.23	1.61	1.61	**0.00**	0.00	0.00	0.00	0.00	0.00	0.00	0.00	0.00	0.00	0.00
**Run length = 3**																				
**New Codes in Run**							2	3	1	1	0	0	0	0	0	0	0	0	0	0
**Saturation Ratio* (%)**							**3.23**	4.84	1.61	1.61	**0.00**	0.00	0.00	0.00	0.00	0.00	0.00	0.00	0.00	0.00

Notes. FG = Focus Group. IN = Interview. *Saturation Ratio = number of new codes in run divided by the number of base codes.
