# Supplementary material for: Veteran families with complex needs: a qualitative study of the veterans’ support system
Source: BMC Health Serv Res. 2022 Jan 15;22:74. doi: 10.1186/s12913-021-07368-2 (PMC8760758; doi:10.1186/s12913-021-07368-2)
Supplement: Supplementary file 5 — Additional file 5. Partners in Recovery peer-reviewed literature [file 12913_2021_7368_MOESM5_ESM.pdf]

Additional file 5. 'Partners in Recovery' peer-reviewed literature

1. Banfield M, Forbes O. Health and social care coordination for severe and persistent mental illness in Australia: A mixed methods evaluation of experiences with the Partners in Recovery program. Int J Ment Health Syst. 2018;12(13):1-13. https://doi.org/10.1186/s13033-018-0194-2
2. Brophy L, Hodges C, Halloran K, Grigg M, Swift M. Impact of care co-ordination on Australia's mental health service delivery system. Aust Health Rev. 2014;38:396-400. https://doi.org/10.1071/AH13181
3. Cheverton J, Janamian T. (2016). The Partners in Recovery program: mental health commissioning using value co-creation. Med J Aust. 2016;204(7):S38-40. https://doi.org/10.5694/mja16.00124
4. Gulliver A, Morse AR, Wilson N, Sargent G, Banfield M. An evaluation of a tailored care program for complex and persistent mental health problems: Partners in Recovery program. J Eval Prog Plan. 2018;68:99-107. https://doi.org/10.1016/j.evalprogplan.2018.03.001
5. Hancock N, Scanlan JN, Gillespie JA, Smith-Merry J, Yen I. Partners in recovery program evaluation: changes in unmet needs and recovery. Aust Health Rev. 2018;42(4):445-52. https://doi.org/10.1071/AH17004
6. Hancock N, Scanlan JN, Honey A, Bundy AC, O'Shea K. Recovery Assessment Scale—Domains and Stages (RAS-DS): its feasibility and outcome measurement capacity. Aust N Z J Psychiatry. 2015;49(7):624-33. https://doi.org/10.1177/0004867414564084
7. Hancock N, Smith-Merry J, Gillespie JA, Yen I. Is the Partners in Recovery program connecting with the intended population of people living with severe and persistent mental illness? What are their prioritised needs? Aust Health Rev. 2017;41(5):566-72. https://doi.org/10.1071/AH15248
8. Hancock N, Smith-Merry J, Jessup G, Wayland S, Kokany A. Understanding the ups and downs of living well: the voices of people experiencing early mental health recovery. BMC Psychiatry. 2018;18(1):121. https://doi.org/10.1186/s12888-018-1703-1

9. Hancock N, Smith-Merry J, McKenzie K. Facilitating people living with severe and persistent mental illness to transition from prison to community: a qualitative exploration of staff experiences. Int J Ment Health Syst. 2018;12:45. https://doi.org/10.1186/s13033-018-0225-z
10. Henderson J, Javanparast S, Baum F, Freeman T, Fuller J, Ziersch A, et al. Interagency collaboration in primary mental health care: lessons from the Partners in Recovery program. Int J Ment Health Syst. 2019;13(37):1-10. https://doi.org/10.1186/s13033-019-0297-4
11. Hurley J, Cashin A, Mills J, Hutchinson M, Graham I. A critical discussion of peer workers: implications for the mental health nursing workforce. J Psychiatr Ment Health Nurs. 2016;23(2):129-35. https://doi.org/10.1111/jpm.12286
12. Hurley J, Cashin A, Mills J, Hutchinson M, Kozlowski D, Graham I. Qualitative study of peer workers within the 'Partners in Recovery' programme in regional Australia. Int J Ment Health Nurs. 2018;27(1):187-95. https://doi.org/10.1111/inm.12308
13. Isaacs AN, Beauchamp A, Sutton K, Maybery D. Unmet needs of persons with a severe and persistent mental illness and their relationship to unmet accommodation needs. Health Soc Care Community. 2019;27(4):e246-56. https://doi.org/10.1111/hsc.12729
14. Isaacs AN, Dalziel K, Sutton K, Maybery D. Referral patterns and implementation costs of the Partners in Recovery initiative in Gippsland: learnings for the National Disability Insurance Scheme. Australas Psychiatry. 2018;26(6):586-9. https://doi.org/10.1177/1039856218759408
15. Isaacs AN, Fenn S. Enabling interagency collaboration in providing comprehensive mental health care: the rise of the care coordinator. Australas Psychiatry.2018;26(4):438-9. https://doi.org/10.1177/1039856218765882
16. Isaacs AN, Firdous F. A care coordination model can facilitate interagency collaboration when designing recovery-oriented services. J Psychosoc Nurs Ment Health Serv. 2019;57(4):38-43. https://doi.org/10.3928/02793695-20181128-01

17. Isaacs AN, Sutton K, Dalziel K, Maybery D. (2017). Outcomes of a care coordinated service model for persons with severe and persistent mental illness: a qualitative study. Int J Soc Psychiatry. 2017;63(1):40-7. https://doi.org/10.1177/0020764016678014
18. Schweizer R, Honey A, Hancock N, Berry B, Waks S, Scanlan JN. Consumer-provider relationships in a care coordination model of service: consumer perspectives. Adv Ment Health. 2018;16(1): 88-100. https://doi.org/10.1080/18387357.2018.1425095
19. Smith-Merry J, Gillespie J. Embodying policy-making in mental health: the implementation of Partners in Recovery. Health Soc Rev. 2016;25(2):187-201. https://doi.org/10.1080/14461242.2016.1171120
20. Smith-Merry J, Gillespie J, Hancock N, Yen I. Doing mental health care integration: a qualitative study of a new work role. Int J Ment Health Syst. 2015;9(32):1-14. https://doi.org/10.1186/s13033-015-0025-7
21. Sutton K, Isaacs AN, Dalziel K, Maybery D. Roles and competencies of the support facilitator in Australia's recovery-oriented mental health initiative: a qualitative study from Gippsland, Victoria. Aust Health Rev. 2017;41(1):91-7. https://doi.org/10.1071/AH15183
22. Trankle SA, Reath J. Partners in Recovery: An early phase evaluation of an Australian mental health initiative using program logic and thematic analysis. BMC Health Serv Res. 2019;19:524. https://doi.org/10.1186/s12913-019-4360-2
23. Waks S, Scanlan JN, Berry B, Schweizer R, Hancock N, Honey A. Outcomes identified and prioritised by consumers of Partners in Recovery: A consumer-led study. BMC Psychiatry. 2017;17:338. https://doi.org/10.1186/s12888-017-1498-5
